# No evidence for viral sequences in five lepidic adenocarcinomas (former “BAC”) by a high-throughput sequencing approach

**DOI:** 10.1186/s13104-015-1669-8

**Published:** 2015-12-15

**Authors:** Nicolas Berthet, Lionel Frangeul, Ken André Olaussen, Elisabeth Brambilla, Nicolas Dorvault, Philippe Girard, Pierre Validire, Elie Fadel, Christiane Bouchier, Antoine Gessain, Jean-Charles Soria

**Affiliations:** Epidemiology and Physiopathology of Oncogenic Viruses Unit, Institut Pasteur, 28 rue du Docteur Roux, 75015 Paris, France; Centre National de la Recherche Scientifique, UMR 3569, 28 rue du Docteur Roux, 75015 Paris, France; Viruses and RNAi Unit, Institut Pasteur, 28 rue du Docteur Roux, 75015 Paris, France; INSERM - U981, 94805 Villejuif, France; Gustave Roussy, DHU TORINO, 94805 Villejuif, Paris, France; Univ Paris-Sud, UMR-S981, 94805 Villejuif, Paris, France; INSERM U823, Institut Albert Bonniot-Université Joseph Fourier, Grenoble Cedex 09, France; Département d’Anatomie et Cytologie Pathologiques, CHU Albert Michallon, BP 217, 38043 Grenoble Cedex 09, France; Département thoracique, Institut Mutualiste Montsouris, 42 Boulevard Jourdan, 75014 Paris, France; Département d’anatomie pathologique, Institut Mutualiste Montsouris, 42 Boulevard Jourdan, 75014 Paris, France; INSERM, U999, 92350 Le Plessis-Robinson, France; Univ Paris-Sud, UMR-S999, 92350 Le Plessis-Robinson, France; Department of Thoracic and Vascular Surgery and Heart-Lung Transplantation, Centre Chirurgical Marie Lannelongue, 92350 Le Plessis-Robinson, France; Plateforme de Génomique, Institut Pasteur, 28 rue du Docteur Roux, 75015 Paris, France; Département Zoonoses et Maladies Emergentes, Groupe Syndromes Cliniques et Virus Associés, Centre International de Recherches Médicales de Franceville (CIRMF), BP 769, Franceville, Gabon

## Abstract

**Background:**

The hypothesis of an infectious etiology of the formerly named bronchiolo-alveolar carcinoma (BAC) has raised controversy. We investigated tumor lung tissues from five patients with former BAC histology using high-throughput sequencing technologies to discover potential viruses present in this type of lung cancer. Around 180 million single reads of 100 bases were generated for each BAC sample.

**Results:**

None of the reads showed a significant similarity for Jaagsiekte sheep retrovirus (JSRV) and no other viruses were found except for endogenous retroviruses.

**Conclusions:**

In conclusion, we have demonstrated the absence of JSRV and other known human viruses in five samples of well-characterized lepidic adenocarcinoma.

**Electronic supplementary material:**

The online version of this article (doi:10.1186/s13104-015-1669-8) contains supplementary material, which is available to authorized users.

## Background

The bronchiolar-alveolar cancer (BAC) in its past definition (WHO classification 1999) is a rare form of lung adenocarcinoma (ADC). The international WHO 2015 classification recommends distinguishing adenocarcinoma in situ (AIS, formerly non-mucinous BAC) from invasive mucinous adenocarcinoma (IMA, formerly mucinous BAC) and non-mucinous lepidic predominant invasive adenocarcinoma of the lungs [1]. In many such patients, the tumor progression respects the pulmonary architecture and develops mainly in the terminal respiratory unit (lepidic growth).

The etiology of these cancers still remains unclear. Interestingly, lung adenocarcinoma cancers with predominant lepidic pattern can be distinguished from other pulmonary non-small-cell carcinomas by an increased frequency of onset in young subjects, women, non-smokers and Asians. Unlike other lung adenocarcinomas, invasive mucinous adenocarcinomas (IMA) the first and more frequent variant of adenocarcinomas (WHO 2015) are multifocal or diffuse and the death is generally due to the bilateral pulmonary spread rather than the onset of metastases.

Given the fact that these cancers are rarely metastatic, a replacement of the cancerous lung by an allograft is possible. Some cases of recurrence of the cancer in a transplanted lung (initially healthy) have led us to question the existence of an infectious agent capable of recolonizing the transplant itself. The hypothesis of an infectious etiology of the formerly named “BACs” has raised controversy, which has been reopened by the observation that the ovine pulmonary adenocarcinoma, which shows some strong clinical and histological similarities with human “BACs”, is associated causally to the infection by the JSRV retrovirus (Jaagsiekte sheep retrovirus) [2–5]. However, in humans, molecular approaches mainly based on PCR technology aiming to reveal the genome of the JSRV virus in the patients with a bronchiolar-alveolar cancer have nevertheless been mostly found negative [6, 7].

The aim of this study was to explore more broadly the hypothesis of a viral etiology of invasive adenocarcinoma with lepidic growth by benefiting from high-throughput sequencing technologies. During the last decade, these approaches have enabled to discover and characterize some new viruses associated to chronic and/or acute human diseases [8, 9]. This is exemplified by the identification in 2008, of the first human oncogenic polyomavirus and its association with the Merkel cell carcinoma, a rare but aggressive skin cancer [8].

## Methods

### Tumor samples

Frozen tumor lung tissues were retrospectively collected from five patients with former “BAC” histology treated at the Institut Mutualiste Montsouris (Paris, France) between 2007 and 2011. Patient characteristics are summarized in Table 1. Briefly, among these, three were female patients, two were never smokers and three were former smokers. As usually observed in this disease, the maximum standardized uptake values (SUVmax) during PET/CT scan examination of the patients showed generally low fixation values. Two of the cases with former BAC diagnosis showed invasive mucinous histology whereas three were non-mucinous. All cases were pathologically reviewed (EB) according to the recent WHO 2015 classification of lung adenocarcinoma. Two supplementary cases of squamous cell carcinoma from two female smokers, obtained at the Centre Chirurgical Marie-Lannelongue (CCML, Le Plessis-Robinson, France) were used as negative controls in the bioinformatic analysis. The study was approved by the local ethics committee and written informed consents were obtained for all patients included.

**Table 1:** Patient characteristics

Patient number	AT1	AT2	AT3	AT4	AT5
Gender	Female	Female	Male	Male	Male
Age (years)	46	76	62	76	66
Smoking status	Never	Former	Former	Never	Former
TEP (SUVmax)	“Weak”	2.5	0	2.6	1.8
TNM	pT1N0	pT2N0	TxN0	pT4N0	pT2bN0
Mucinous	No	Yes	No	No	Yes
Pathological analysis	NMLIA	IMA	Mixed AIS and minimally invasive ADC	NMLIA	IMA

*AT* adenocarcinoma-tumor, *TEP/SUVmax* TEP/standardized uptake values, *ADC* adenocarcinoma, *AIS* adenocarcinoma in situ, *IMA* invasive mucinous adenocarcinoma, *NMLIA* non mucinous lepidic invasive adenocarcinoma

### Samples associated with known human oncogenic viruses

The sensibility of the high-throughput sequencing assay used here has been evaluated in a pilot study thanks to the detection of HTLV-1 and HHV-8, two very different known oncogenic viruses associated causally to the adult T cell leukemia/lymphoma (ATLL) for HTLV-1 and to the primary effusion lymphoma (PEL) for HHV-8. These two oncogenic viruses were chosen because they are quite different according to their size (around 9 kb for HTLV-1 vs 200 kb for HHV-8), for their genomic form in the cancer cells (integrated in the genome DNA for HTLV-1 and latent episomal for HHV-8) and more importantly for their number of copies present in the tumors. Indeed, in ATLL, one or two copies of HTLV-1 viral genome are integrated into each cancer cells whereas the viral load is much higher in PEL with around 100–300 HHV-8 copies by cell. Two frozen samples of uncultured ex vivo tumors cells were used. The first one originated from an ATLL associated with HTLV-1, the second one was a PEL associated with HHV-8. The preparation of the nucleic acid, the sequencing process and the bio-informatics analyses are described in the Additional file 1.

### Methodology of data analysis

**Fig. 1:**
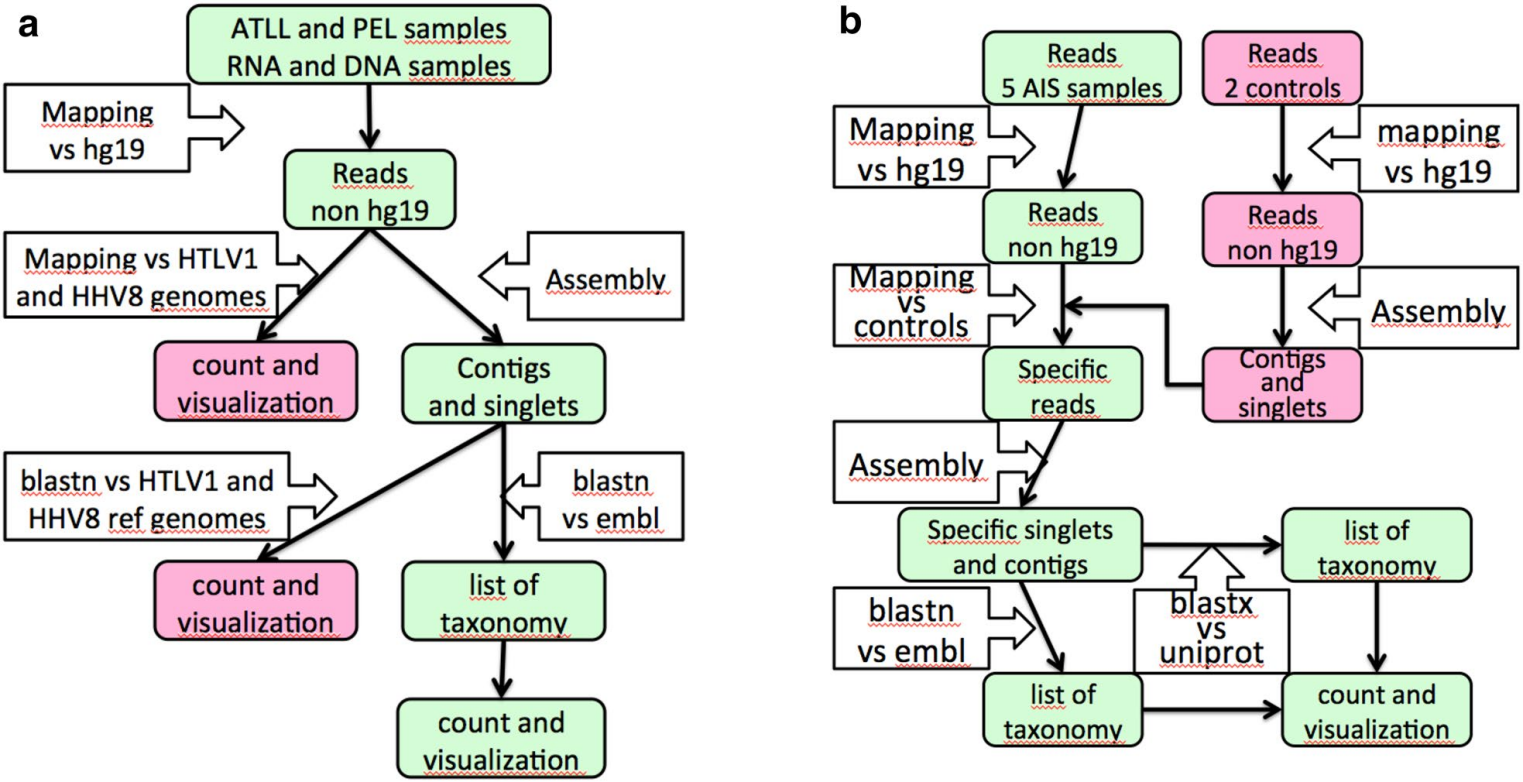
Methodology used for the pilot study (**a**) and the main study (**b**). The common process for the two studies is summarized in the *green* pathway. The specific control processes for each study are summarized in the *pink* pathways

Data generated for the pilot study on ATLL and PEL, on one hand, and for the five adenocarcinoma samples, on the other hand, were subjected to a series of analyses described in Fig. 1. These analyses are distributed between a common process, described in green and applied to both studies, and control processes, outlined in pink, specific to each study. The purpose of the common process was to assign taxonomies to the reads coming from each sample. The count of these taxonomies should help to identify (1) the expected viruses for the pilot study, (2) an infectious agent present in AIS nucleic acid in the main study. More details about the methodology of analysis are given in an Additional file 1.

## Results

For each of the nine samples (the ATLL and the PEL for the pilot study and the five AIS), around 180 million single reads of 100 bp were obtained while around 150 million were obtained for the two for RNA samples (the ATLL and the PEL). The quality of these reads was verified and considered very good for all samples. The reads of each sample were filtered against the human genome (hg19).

## Pilot study: sensibility of the detection of two known oncogenic viruses (HHV-8 and HTLV-1) in ex vivo tumor samples

### Control specific process

The presence of both HTLV-1 and HHV-8 was searched in each of the four studied samples (Table 2). This was done either by mapping reads to reference genomes using bowtie2 [10], or by searching a similarity between the contigs/singlets and the reference genomes using blastn [11] (Table 2; Fig. 1a). Concerning the PEL sample, numerous reads originating from the DNA or the RNA corresponded to the HHV-8 reference genome. Indeed, a total of 176,000 (31 %) to 228,000 (45 %) reads were identified as deriving from the HHV-8 genome (Table 2). In contrast, in this PEL sample, not a single HTLV-1 read was found (Table 2). The HHV-8 genome is covered at 85–92 % by the contigs stem from the assembly step (Additional file 1: Figures S1 and S2). Concerning the ATLL sample, the results were very different as only 215 (0.06 %) and 312 (0.14 %) reads were identified, as originating from the HTLV-1 genome, in the DNA and RNA samples respectively. Contigs from these samples cover only 45–60 % of the retroviral genome (Additional file 1: Figures S3 and S4).

**Table 2:** Results of the control and the common processes of the pilot study on the PEL and ATLL samples ex-vivo samples

		Pilot study			
		PEL		ATLL	
	Samples	HHV-8 RNA	HHV-8 DNA	HTLV-1 RNA	HTLV-1 DNA
HiSeq2000 run	Total reads	144,170,345	118,055,719	171,301,521	154,800,548
Quality and hg19 filters	Remaining reads	500,318	566,619	226,120	375,503
Control process					
Mapping (bowtie2)	Nber of reads vs HHV-8 genomes	227,842 (45 %)	175,905 (31 %)	0 (0 %)	0 (0 %)
	Nber of reads vs HTLV-1 genomes	0 (0 %)	0 (0 %)	321 (0.14 %)	215 (0.06 %)
Similarity (blastn)	Contigs similar to HHV-8. Genome covered by contigs (%)	22/1412 contigs (85 %)	11/5080 contigs (92 %)	0/1112 contigs (0 %)	0/5463 contigs (0 %)
	Contigs similar to HTLV-1. Genome covered by contigs (%)	0/1412 contigs (0 %)	0/5080 contigs (0 %)	12/1112 contigs (60 %)	9/5463 contigs (45 %)
Common process					
Taxonomic assignation (blastn)	Contigs without taxonomy. Total length	118/1412 (8 %), 21,807 bases (4.5 %)	615/5080 (12 %), 173,474 bases (9 %)	141/1112 (13 %), 25,388 bases (9 %)	698/5463 (13 %), 214,796 bases (10 %)
	Taxonomy count	41 % viruses including 100 % Rhadinovirus	80 % viruses including 100 % Rhadinovirus	0.7 % viruses including 98 % HTLV-1	0.08 % viruses including 86 % HTLV-1

### Common process

The taxonomic assignment performed by BLASTN (against the EMBL database) on the contigs (similar to those used for above in the control process) of each sample demonstrated the ability of this process to identify the two viruses. For the PEL sample, HHV-8 presence was obvious since it represents 41 % to 80 % of assignments in the RNA and DNA samples respectively, after filtering. The background of the analysis is minimal since the taxonomy “Rhadinovirus” represents 100 % of viral taxonomies (Additional file 1: Figures S5 and S6). For ATL, HTLV-1 was represented by only a few number of small contigs, however, the analysis process also leads to the detection of its presence. Although viral taxonomies represent only 0.08–0.7 % of the assignments (Additional file 1: Figures S7 and S8), in this viral branch, HTLV-1 represents 86–98 % of the reads (Additional file 1: Figures S9 and S10). These differences between HHV-8 and HTLV-1 results were in agreement with the theoretical prediction that takes into account the size of the two genomes and their number of copies per cell.

In conclusion, long contigs representing almost all the viral genome can be obtained with the reads generated with the DNA (Additional file 1: Figures S1–S4). The RNA data bring more information on the expression of the viral genes in the tumor but are less “informative” for its detection and characterization because only small contigs representing small parts of the genome are obtained. Based on these data, the use of genomic DNA in the IMA study was considered as a good approach for the research of a potential infectious agent present in these tumors, especially if we consider that such agent could be a DNA virus. Indeed, up to know, almost all oncogenic viruses are DNA viruses with the exception of HCV and HTLV-1.

### Adenocarcinoma study

Similarities were initially searched using blastn, between some known viruses and all reads of each sample without any prior filtering, especially against hg19. These viruses were the Jaagsiekte sheep retrovirus (JSRV, Acc NC_001494.1), the Human herpesvirus 6B (HHV6B, Acc AB021506), Epstein–Barr virus (EBV, Acc M80517) and the Human endogenous retrovirus K113 (HERV-K113, Acc AY037928). None of the reads in the five samples showed a significant similarity for JSRV, HHV6 and EBV. In contrast and, as expected, HERV was present in all the samples, with more than 50,000 reads per samples overlapping the entire HERV genome.

For *control process*, the filtering of five tumor samples allowed us to significantly reduce the number of human reads and contaminants (Table 1). Indeed, 62–91 % of the reads, remaining after hg19 filtering, were removed by this second filter using the two control samples (Table 3). Although the percentage of filtered reads is high, the number of remaining reads for each sample were variable with a maximum range from 0.3 × 10^5^ reads for sample AT5 to 2 × 10^5^ for sample AT1 (Table 3).

**Table 3:** Results of the control and the common process of the main study. Filters, assembly and taxonomic assignment of five patient tumor samples with former BAC histology (AT1–5)

Quality and hg19 filters			Main study					
		Samples	AT1	AT2	AT3	AT4	AT5	5 samples AT1-5
		Remaining reads	579,994	618,449	619,414	613,570	433,398	2,864,825
Control process								
Mapping (bowtie2)		Nber of reads vs control samples	357,616 (62 %)	407,370 (66 %)	555,964 (89 %)	476,430 (78 %)	396 ,251 (91 %)	2,193,631 (76 %)
		Remaining reads	222,378	211,079	63,450	137,140	37,147	671,194
Common process								
Global assembly (SPAdes)		Contigs with reads from this sample	269 (45 %)	307 (51 %)	278 (46 %)	324 (54 %)	285 (48 %)	596 (100 %)
		Contigs only with reads from this sample	23 (3.8 %)	41 (6.8 %)	31 (5.2 %)	32 (5.3 %)	57 (9.5 %)	45 (7.5 %)
Taxonomic assignation (blastn)		Contigs without taxonomy. Total length	54 contigs 4599 bases	66 contigs 13,688 bases	56 contigs 10,662 bases	70 contigs 15,426 bases	74 contigs 16,785 bases	162 contigs 36,852 bases (20 %)
		Taxonomy count	NA	NA	NA	NA	NA	96 % Eukaryota including 76 % Hominidae. 4 % vectors

An overall assembly of all these filtered reads allowed us to obtain 596 contigs (global assembly Table 3). Similarities between these contigs and the sequences of the EMBL database (http://www.ebi.ac.uk/ena/home) were searched for using blast. A total of 434 significant similarities led to a taxonomic assignment for the same number of contigs. These taxonomies, depicted by the krona software [12] were overwhelmingly eukaryotic (96 %) and more specifically hominidae (76 %) (Additional file 1: Figure S11). The remaining sequences consist of contigs, shorter than 200 bases, with small similarities (30–40 bases) with sequences annotated as vectors (in green in Additional file 1: Figure S11). The taxonomic assignment provided by blastx against Uniprot databases (http://www.uniprot.org/) were almost identical (data not shown).

For 16 contigs, all from the AT2 sample, taxonomic assignment obtained by similarities close to 100 % is unexpected. These taxonomies refer to sequences from Mus musculus and never described in human sequences. A total of 12 contigs covering the entire CDS and the 3′ untranslated region of a LINE-1 sequence (L1spa Acc AF016099.1, L1Md-tf23 Acc AF081110); three contigs partially overlap the Gag/pol region of an intracisternal A-particle sequence (MIA14, Acc M17551.1) and one contig, the only one with high coverage, is identical to a sequence described as a major satellite repeat sequence (Acc EF028077). To our knowledge, these three sequences have not been interconnected in a database sequence or in a publication but all three are annotated as mobile elements.

## Discussion and conclusion

The goal of our study was to search for the presence of a virus in well-characterized ex vivo samples of human lung adenocarcinoma, especially in the bronchiolar-alveolar subtype. We specifically focused our study on these relatively rare tumors because: (1) Based on clinical aspects, the hypothesis of an infectious etiology has been raised in such tumors. (2) The ovine pulmonary adenocarcinoma which exhibits some similarities with mucinous BAC (now IMA) is considered causally associated with the JSRV retrovirus. (3) The published data concerning the detection by molecular means and/or by immuno-histochemistry of such a retrovirus in mucinous BAC still remained controversial. Indeed, the most recent report focusing specifically on the JSRV candidate in histological sections of different lung cancers, including BAC, found minor evidence of JSRV *env*-specific immunoreactivity and JSRV-like *env* and *gag* sequence amplification. The authors suggest that a JSRV-like virus might infect human lungs and have oncogenic properties in several subtypes of lung cancer [13]. Conversely, other studies have failed at showing any association between lung cancer and JSRV [6, 7, 14]. Moreover, a large study recently investigated 225 cases of lung adenocarcinomas using RNA-Seq found no DNA virus transcript [15].

Thus, firstly in order to try to resolve the above-discussed controversy concerning the association of JSRV with mucinous BAC and secondly to uncover in such specific lung tumors any other known or unknown viruses, we used the high-throughput sequencing method. Such assay is based on random sequencing of nucleic acid present in a given sample. This very broad approach method is highly powerful, and can detect any known or unknown agent with a sensitivity level roughly equivalent to current specific PCR methods [16]. Furthermore, this technological approach with a sufficient depth of sequencing, as performed here, is able to confirm the absence of a suspected infectious agent in targeted pathologies [15, 17, 18]. Lastly, a previous study showed the effectiveness of such a method on samples artificially infected with low levels of known viruses [16].

The first step of the study was to show the effectiveness of the use of our methodology for two human cancer (ATLL or PEL) samples naturally infected by two different oncogenic viruses respectively (HTLV-1 and HHV-8). The entire pilot project demonstrated a high sensitivity to our approach, which was thus subsequently applied to BAC samples. Indeed, our method allowed us to detect but also identify nearly the entire sequence, or large portions, of these two known viral genomes in the specific cancer samples. Furthermore, our data also showed good specificity with, on one hand, a low background noise at both viral and bacterial level and, on the other hand, a lack of contamination between samples. Indeed, none of the HHV-8 reads could be detected by bowtie2 or by BLASTN in the ATLL samples despite the fact that the samples were handled together and sequenced in the same flow cell.

Our study has several limitations:

1. This study was conducted in only 5 European patients.
2. There were no AIS (pure lepidic growth pattern) included in this study.
3. Some interesting microbial sequences, especially of viral origin but wrongly considered as belonging to (or assigned to) *Homo sapiens*, could have been eliminated through the filtering process.
4. A relatively high number of contigs (162), representing more than 300,00 bp, remained without any taxonomic assignation at the end of our analyses. It could be hypothesized that one, or few of them, could correspond to a part of a yet unknown infectious agent. Clarifying this situation will need further in depth studies searching for possible viral specific structure in such contigs.
5. Finally, the absence of any viral sequences in the findings could be linked to the possibility of a very low viral load of the searched virus in only a small proportion of the tumor cells. Such unlikely situation has however been raised in some cases of Merkel cell carcinoma [8].

In conclusion, we have demonstrated the absence of JSRV and other known human viruses, before and after any filtering, in these five samples of well-characterized lepidic adenocarcinomas.

## Additional file

10.1186/s13104-015-1669-8 Description of bioinformatic analysis and supplementary results for pilot and patient studies.

## Abbreviations

IASLC/ATS/ERS: International Association for the Study of Lung Cancer/American Thoracic Society/European Respiratory Society
ADC: adenocarcinoma
BAC: bronchiolar-alveolar cancer
AIS: adenocarcinoma in situ
IMA: invasive mucinous adenocarcinoma
NMLIA: non mucinous lepidic invasive adenocarcinoma
EGFR: epidermal growth factor receptor
KRAS: v-Ki-ras2 Kirsten rat sarcoma viral oncogene homolog
JSRV: Jaagsiekte sheep retrovirus
SUV: standardized uptake value
PET/CT: positron emission tomography–computed tomography
HTLV-1: human T-lymphocyte virus type 1
HHV-8: human herpesvirus type 8
ATL: adult T cell leukaemia/lymphoma
PEL: primary effusion lymphoma
HCV: hepatitis C virus
HERV: human endogen retrovirus
BLAST: basic local alignment search tool
LLA: lepidic lung adenocarcinoma
