# Supplementary material for: No evidence for viral sequences in five lepidic adenocarcinomas (former “BAC”) by a high-throughput sequencing approach
Source: BMC Res Notes. 2015 Dec 15;8:782. doi: 10.1186/s13104-015-1669-8 (PMC4678645; doi:10.1186/s13104-015-1669-8)

**Supplemental Methods**

Preparation of nucleic acid (DNA/RNA) and sequencing.

Ten cryostat sections (20-µm) of each tumor sample were submitted to nucleic acid extraction. Presence of at least 50% tumor cells was verified in a light microscope using standard HES-staining. They were initially grinded with the cell lyser and the nucleic acids were extracted with the RNAeasy microkit according to the manufacturer’s instructions (Qiagen GmbH, Hilden, Germany). The extracted RNA was treated with TurboDNase (Invitrogen Inc., Carlsbad, CA) and then retrotranscribed into cDNA using SuperScript III reverse transcriptase (Invitrogen Inc., Carlsbad, CA) and random hexamer primers. The cDNA was amplified based on Phi29 polymerase as previously described [16]. The different extracted DNAs were directly sequenced without prior amplification whereas the extracted RNAs were amplified after a depletion step intended to eliminate the maximum of ribosomal RNA. The Illumina Sequencing using HiSeq 2000 was conducted with a mean depth per sample of 1.5×10^8^ single reads of 100 nucleotides (nt) size. For the ATLL sample, the DNA and the RNA runs produces 1.54 and 1.71x10^8^ reads respectively. For the PEL sample, the DNA and the RNA runs produces 1.31 and 1.44x10^8^ reads respectively whereas a mean depth per AIS sample of 1.8.x10^8^ (range 1.5–1.9×10^8^) single reads of 100 nucleotides (nt) size was obtained.

Pilot Study.

The quality of the reads was assessed by FastQC (web site: http://www.bioinformatics.babraham.ac.uk/projects/fastqc/) then the sequences were selected and trimmed according to their quality scores with cutadapt (web site : https://code.google.com/p/cutadapt/). The human genome was filtered by mapping the reads on the Homo sapiens hg19 reference with bowtie2 using the "sensitive" flag option [17]. For the RNA runs, the percentage of reads mapped on the rRNA was 8.6% for HTLV1 sample and 25% for the HHV8 sample. Bowtie2 was used on the remaining reads to search there similarity with the two reference genomes (HTLV1 Acc J02029 and HHV8 Acc AF148805) then the reads were assembled with SPAdes [18] to produce 4 set of contigs.

Patient study.

The quality of the reads was assessed by FastQC then the sequences were selected and trimmed according to their quality scores with cutadapt. The human genome was filtered by mapping the reads on the Homo sapiens hg19 reference with bowtie2 using the "sensitive" flag option. This host filtering step eliminated an average of 99.5% reads per sample (range 99.4-99.7%). The remaining reads of the two control samples were assembled with SPAdes [18] to produce a set of control-contigs (43714 contigs with 5.7X106 bases). For the five other samples, the remaining sequences were further filtered by mapping against the control-contigs using bowtie2 ("sensitive" parameters). This second filtering step eliminated an average of 76% reads per sample (range 61-91%).

At the end of the filtering steps, each sample consisted of a set of sequences containing between 0.3 and 2.2x10^5^ reads. These groups of reads were assembled individually and mixed together by SPAdes assemblers [18]. The mixed assembly produce by SPAdes was composed of 596 contigs with an average length of 312 bases (maximum length = 2982 bases). For all contigs and singlets an attempt at taxonomic assignment has been made ​​by similarity search with sequences of EMBL database (STD section) using BLASTN [19] and with sequences of Uniprot database using BLASTX with an E-value equal to 1E-3. All the possible assignments were counted with a weight equivalent to the number of reads of each contig. The results were sorted by homemade software (taxoptimizer/rankoptimizer) and explored using the krona visualization system [20].

**Supplemental figures.**

**Figure S1**. Results of the control processes for the RNA library from the HHV8 sample (pilot study). **A**: Annotation of the HHV8 genome (Acc AF148805). **B1**: Bowtie2 mapping of the RNA library, reads used in sens are drawn in red ; reads used in anti-sens are drawn in green. The most expressed genes are noted (mainly K proteins). **B2** : zoom of the same mapping. **C**: blastn mapping of the contigs built with the RNA library.


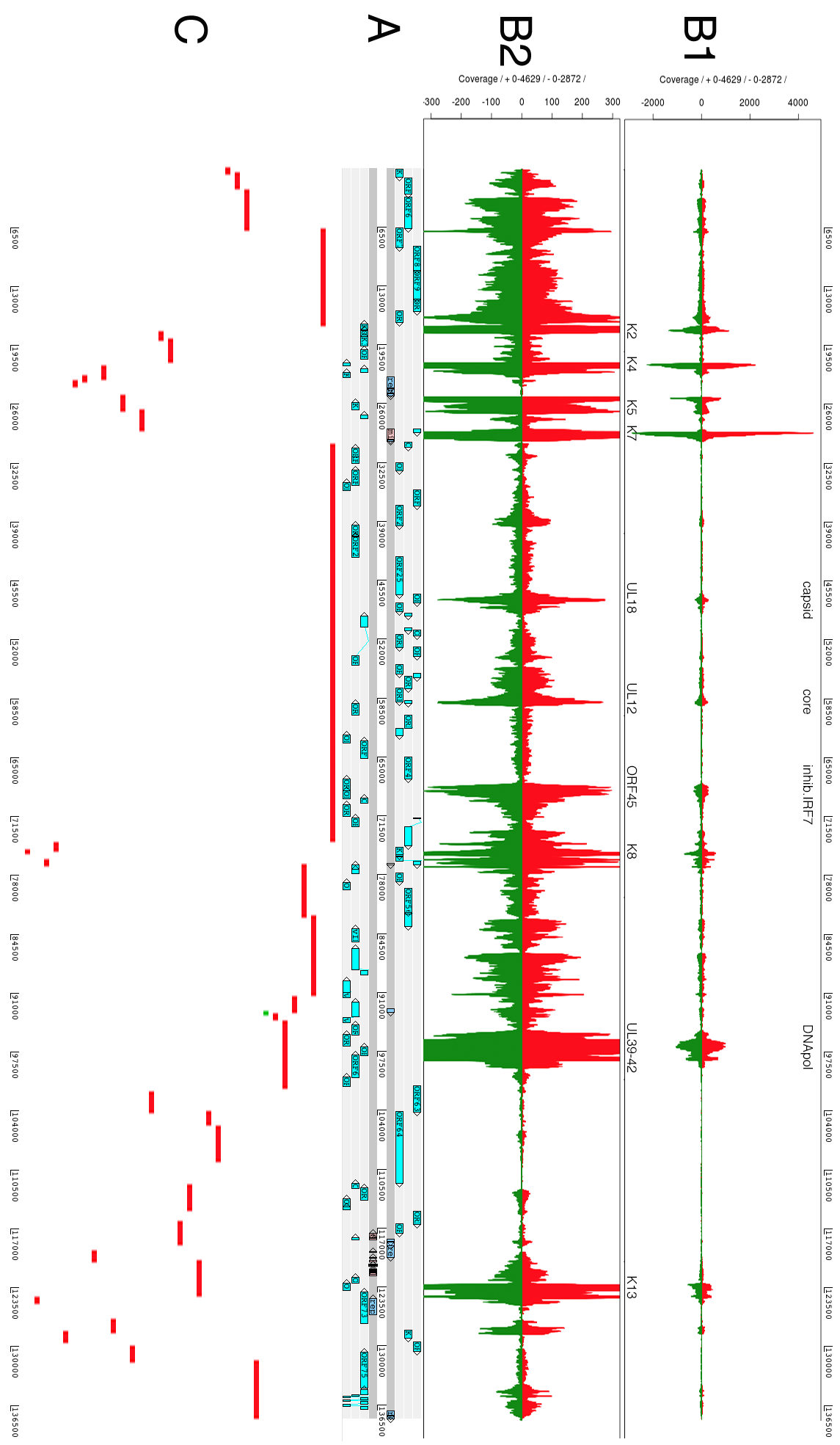


**Figure S2**. Results of the control processes for the DNA library from the HHV8 sample (pilot study). **A**: Annotation of the HHV8 genome (Acc AF148805). **B**: Bowtie2 mapping of the DNA library, reads used in sense are drawn in red ; reads used in anti-sense are drawn in green. **C**: blastn mapping of the contigs built with the DNA library.


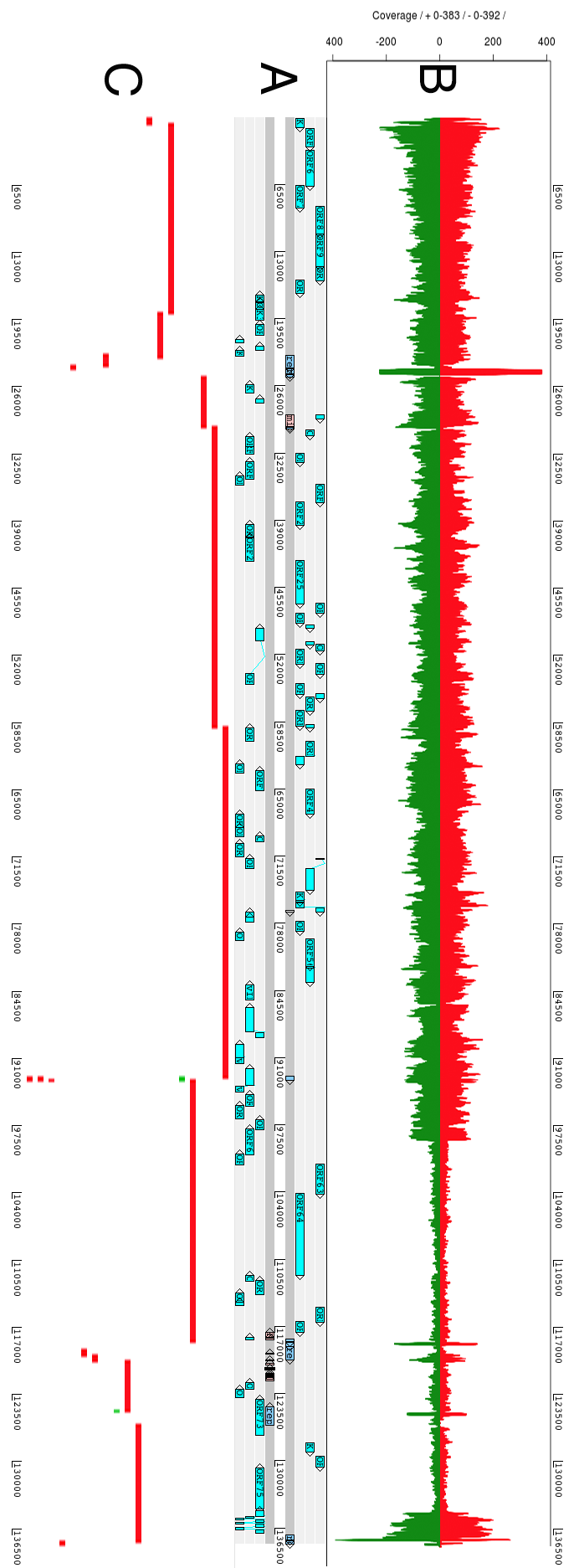


**Figure S3**. Results of the control processes for the RNA library from the HTLV1 sample (pilot study). **A**: Annotation of the HTLV1 genome (Acc AJ02029). **B**: Bowtie2 mapping of the RNA library, reads used in sense are drawn in red ; reads used in anti-sense are drawn in green. **C**: blastn mapping of the contigs built with the RNA library.


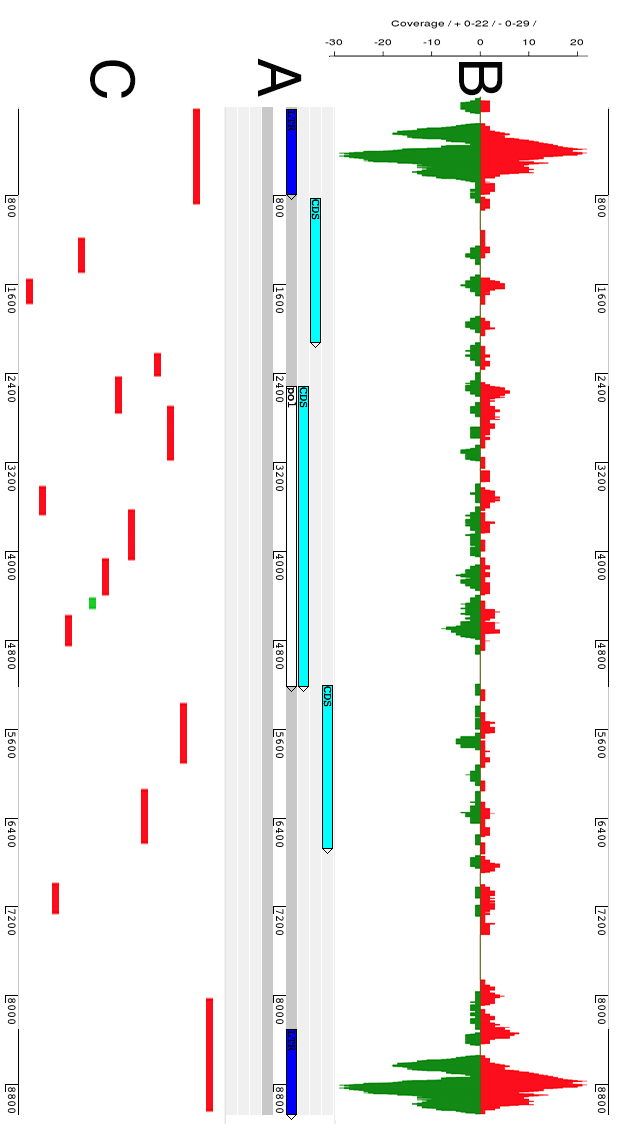


**Figure S4**. Results of the control processes for the DNA library from the HTLV1 sample (pilot study). **A**: Annotation of the HTLV1 genome (Acc AJ02029). **B**: Bowtie2 mapping of the DNA library, reads used in sense are drawn in red ; reads used in anti-sense are drawn in green. **C**: blastn mapping of the contigs built with the DNA library.


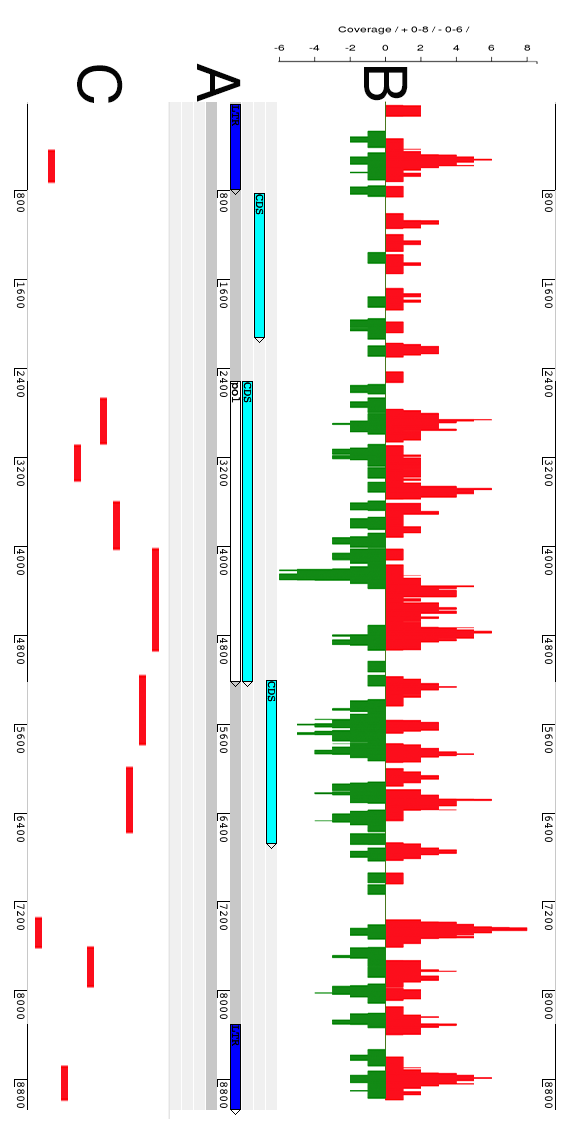


**Figure S5**. Krona representation of the taxonomies incidence from the RNA library of the HHV8 sample.


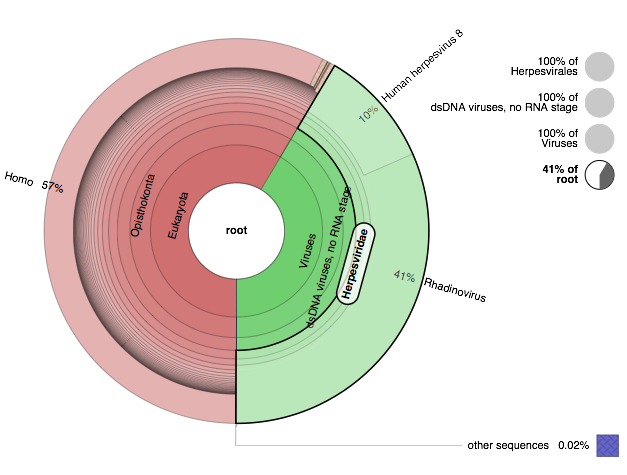


**Figure S6**. Krona representation of the taxonomies incidence from the DNA library of the HHV8 sample.


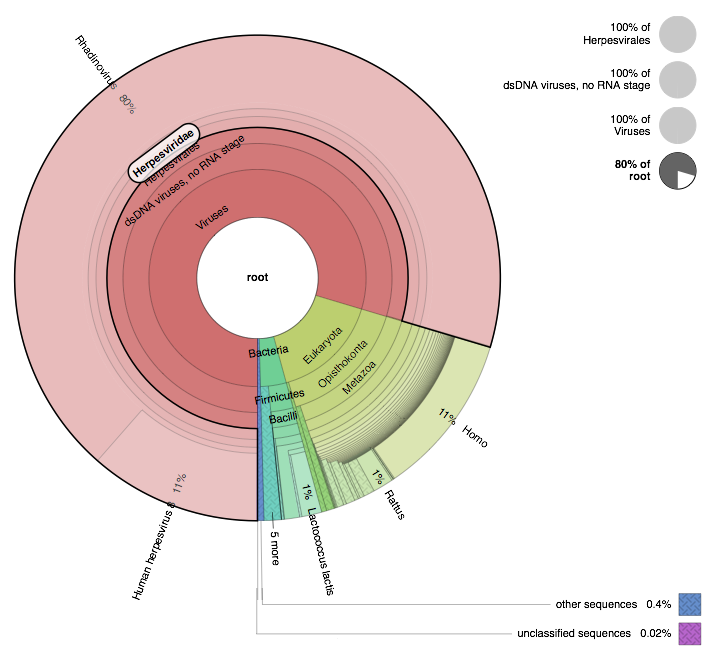


**Figure S7**. Krona representation of the taxonomies incidence from the RNA library of the HTLV1 sample.


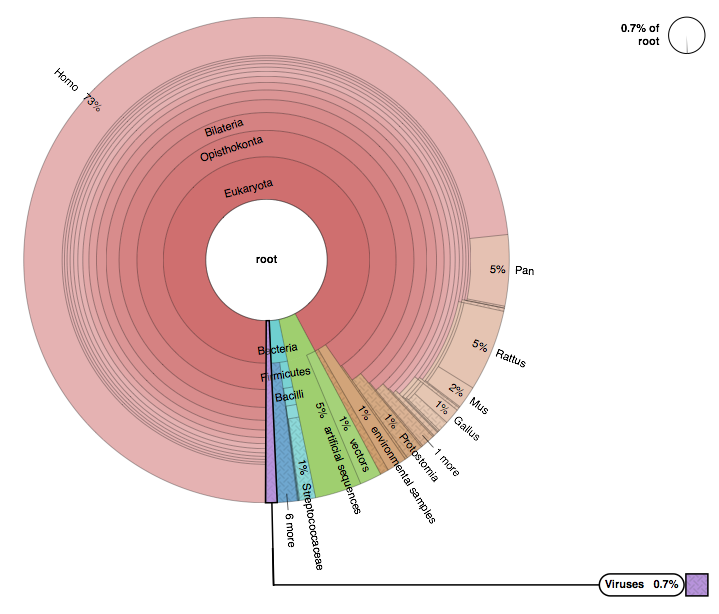


**Figure S8**. Krona representation of the taxonomies incidence for the branch "Viruses" from the RNA library of the HTLV1 sample.


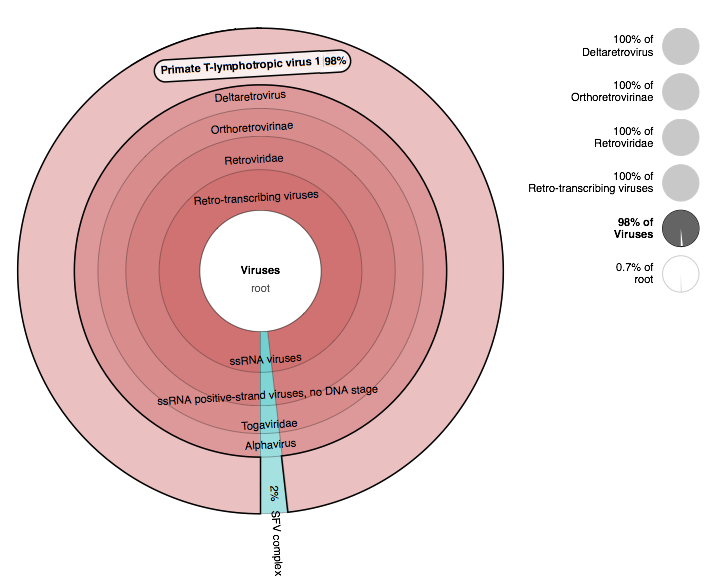


**Figure S9**. Krona representation of the taxonomies incidence from the DNA library of the HTLV1 sample.


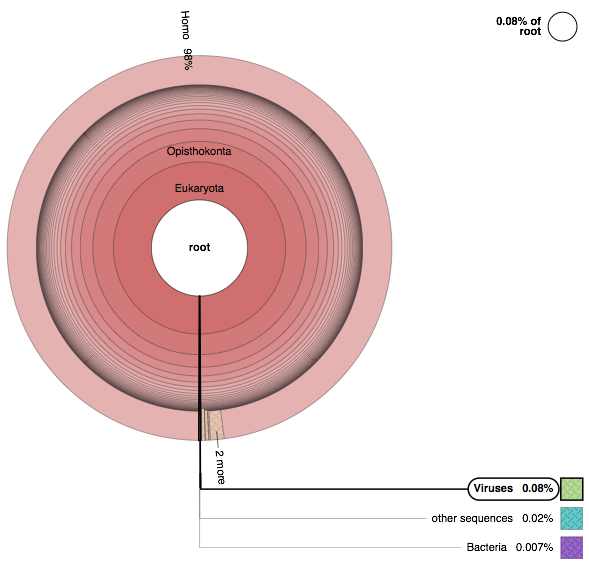


**Figure S10**. Krona representation of the taxonomies incidence for the branch "Viruses" from the DNA library of the HTLV1 sample.


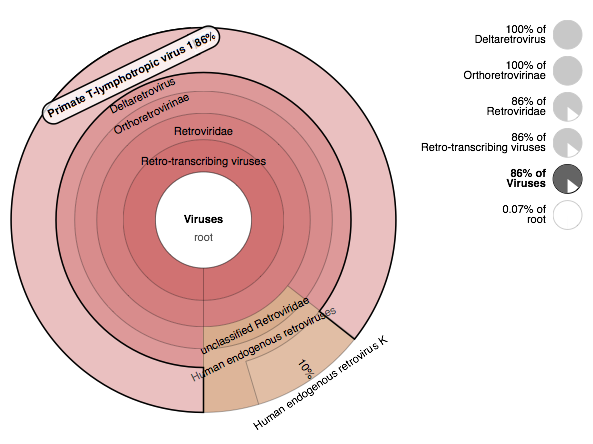


**Figure S11**. Krona representation of the taxonomies incidence from global assembly of the 5 BAC samples.


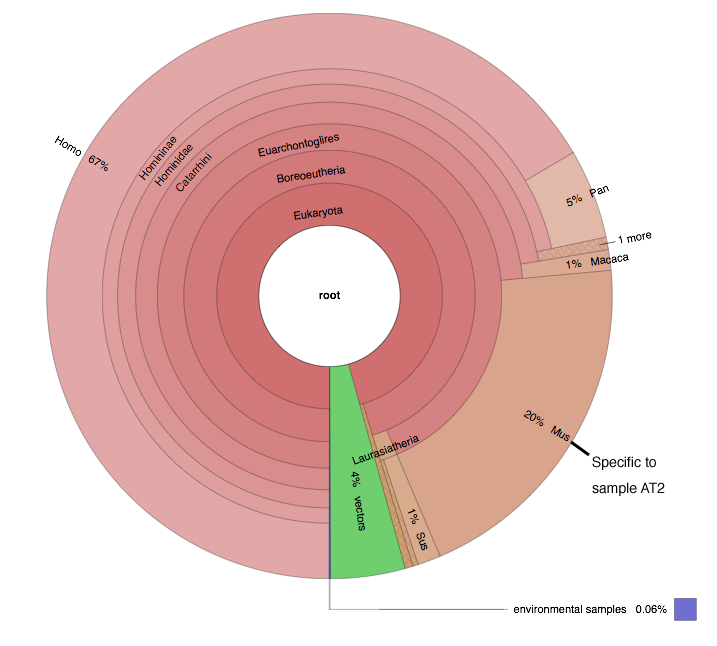

Supplement: Supplementary file 1 — 10.1186/s13104-015-1669-8 Description of bioinformatic analysis and supplementary results for pilot and patient studies. [file 13104_2015_1669_MOESM1_ESM.docx]
